# A Green Algae Mixture of *Scenedesmus* and *Schroederiella* Attenuates Obesity-Linked Metabolic Syndrome in Rats

**DOI:** 10.3390/nu7042771

**Published:** 2015-04-14

**Authors:** Senthil Arun Kumar, Marie Magnusson, Leigh C. Ward, Nicholas A. Paul, Lindsay Brown

**Affiliations:** 1 Centre for Systems Biology, University of Southern Queensland, Toowoomba 4350, Australia; 2 MACRO—the Centre for Macroalgal Resources & Biotechnology, College of Marine and Environmental Sciences, James Cook University, Townsville 4811, Australia; E-Mails: marie.magnusson@jcu.edu.au (M.M.); nicholas.paul@jcu.edu.au (N.A.P.); 3 School of Chemistry and Molecular Biosciences, The University of Queensland, Brisbane 4072, Australia; E-Mail: l.ward@uq.edu.au; 4 School of Health and Wellbeing, University of Southern Queensland, Toowoomba 4350, Australia

**Keywords:** visceral obesity, metabolic symptoms, microalgae, insoluble fibre

## Abstract

This study investigated the responses to a green algae mixture of *Scenedesmus dimorphus* and *Schroederiella apiculata* (SC) containing protein (46.1% of dry algae), insoluble fibre (19.6% of dry algae), minerals (3.7% of dry algae) and omega-3 fatty acids (2.8% of dry algae) as a dietary intervention in a high carbohydrate, high fat diet-induced metabolic syndrome model in four groups of male Wistar rats. Two groups were fed with a corn starch diet containing 68% carbohydrates as polysaccharides, while the other two groups were fed a diet high in simple carbohydrates (fructose and sucrose in food, 25% fructose in drinking water, total 68%) and fats (saturated and *trans* fats from beef tallow, total 24%). High carbohydrate, high fat-fed rats showed visceral obesity with hypertension, insulin resistance, cardiovascular remodelling, and nonalcoholic fatty liver disease. SC supplementation (5% of food) lowered total body and abdominal fat mass, increased lean mass, and attenuated hypertension, impaired glucose and insulin tolerance, endothelial dysfunction, infiltration of inflammatory cells into heart and liver, fibrosis, increased cardiac stiffness, and nonalcoholic fatty liver disease in the high carbohydrate, high fat diet-fed rats. This study suggests that the insoluble fibre or protein in SC helps reverse diet-induced metabolic syndrome.

## 1. Introduction

High energy diets with increased saturated fats and refined carbohydrates increase the development of both intra-abdominal obesity and metabolic risk factors such as impaired glucose tolerance, insulin resistance, increased systolic blood pressure, dyslipidaemia, endothelial dysfunction and cardiovascular complications [1,2,3,4]. A healthy dietary pattern including the intake of vegetables, fruits and whole grain fibre as part of energy restriction prevents obesity, promotes weight loss in obese patients, and reverses metabolic risk factors [5,6,7,8]. In addition to physical activity, obesity management includes healthy eating with consumption of food low in total dietary fats, especially saturated and *trans* fats, and low in refined carbohydrates, but high in proteins and fibre, as a long-term lifestyle intervention [9,10].

Microalgae have been considered as part of a healthy diet as they contain omega-3 fatty acids (eicosapentaenoic acid (EPA) and docosahexaenoic acid (DHA)), proteins, amino acids, pigments, vitamins and minerals [11,12]. Microalgae also serve as a reservoir of biologically active compounds, presenting unique structures and functions [13]. Potentially active ingredients including β-carotene, astaxanthin, lutein, phycobiliproteins and insoluble fibre extracted from *Chlorella*, *Spirulina*, *Dunaliella*, *Porphyridium*, and *Scenedesmus* species have been reported for their antioxidant, cardioprotective, hepatoprotective, anti-inflammatory and anti-hyperlipidaemic effects [14,15,16].

Both *Chlorella* and *Spirulina* are commercially-available microalgae that have been examined for their biological effects in attenuating metabolic risk factors such as obesity, type 2 diabetes, insulin resistance, hypertension, dyslipidaemia and non-alcoholic fatty liver disease [17,18,19,20]. *Scenedesmus* species are also common freshwater green microalgae, well-known for their nutritional value and considered as potential food additives [21] but for which commercial production is lacking. It is difficult, however, to maintain monocultures of microalgae in large-scale culture in open systems, and consequently the successful long-term production of microalgae is often based upon using a mixture of species managed instead as a polyculture [22]. This study has therefore used a microalgal mixture of, predominantly, *Scenedesmus dimorphus* and *Schroederiella apiculata,* grown under commercial conditions in polyculture. We have then investigated in rats whether the consumption of this microalgal mixture can reverse the signs of the metabolic syndrome induced by a high carbohydrate and high fat diet.

## 2. Experimental Section

### 2.1. Microalgal Sources and Nutritional Components

*Scenedesmus dimorphus* and *Schroederiella apiculata* (SC) as a microalgal mixture were cultured at the MBD Energy Research and Development facility at James Cook University, Townsville, Australia. All biomass was produced in large outdoor tanks with capacities of >10,000 L.

Biomass was harvested on two occasions separated by ~12 months from 2011 to 2012. At each time, the biomass was immediately freeze-dried, after which sub-samples (~200 g dry weight) were taken for analyses of fibre components, minerals and fatty acid concentrations.

The % nitrogen in the algal biomass was measured by elemental analysis (OEA Labs Ltd., Callington, Cornwall, UK), and subsequently used to calculate the % protein in the biomass as %*N* multiplied by a protein conversion factor of 5.13 for green algae [23]. Soluble and insoluble fibre were analysed on a combined sample of 100 g for each species containing 50 g from each harvest time. Fibre analyses were run using enzymatic-gravimetric methods by Grain Growers, Sydney, Australia (NATA Accred. 66; AOAC Official Method 993.19 for soluble fibre; AOAC Official Method 985.29 for insoluble fibre). Fatty acids were extracted and quantified as described previously [24]. In each sub-sample, 24 trace elements were quantified and mean values are reported (*n* = 2 sub-samples per species). Al^3+^, Ca^2+^, K^+^, Na^+^, S^2−^ and P^3−^ were analysed by Inductively Coupled Plasma Optical Emission Spectrometry, while metals and metalloids (As^2−^, B^+^, Ba^2+^, Cd^2+^, Co^+^, Cr^3+^, Cu^2+^, Fe^3+^, Hg^2+^, Mg^2+^, Mn^2+^, Mo^+^, Ni^2+^, Pb^2+^, Se^2+^, Sr^2+^, V^2+^ and Zn^2+^) were analysed by Inductively Coupled Plasma Mass Spectrometry at the Advanced Analytical Centre, James Cook University, Townsville. All remaining freeze-dried biomass was stored in vacuum-sealed bags under refrigeration until incorporation in the diet.

### 2.2. Rats and Diets

The experimental groups consisted of a total of 48 male Wistar rats (9–10 weeks-old; 336 ± 2 g) supplied by The University of Queensland Biological Resources unit and individually housed in a temperature-controlled (20 ± 2 °C), 12 h light/dark cycle environment with unrestricted access to food and water at the University of Southern Queensland Animal House. The preparation and macronutrient composition of basal diets, including the dietary fatty acid profiles, have been described [25,26]. All experimentation was approved by the Animal Experimentation Ethics Committees of The University of Queensland and the University of Southern Queensland under the guidelines of the National Health and Medical Research Council of Australia.

The rats were randomly divided into 4 separate groups (*n* = 12 each) and fed with corn starch (C), corn starch with 5% *Scenedesmus dimorphus* and *Schroederiella* *apiculata* mixture (CSC), high carbohydrate, high fat (H), high carbohydrate, high fat with 5% *Scenedesmus dimorphus* and *Schroederiella* *apiculata* mixture (HSC). The microalgal mixture-supplemented diets were prepared by adding 5% of freeze-dried microalgal mixture to replace an equivalent amount of water in the diet. The drinking water in H and HSC rats included 25% fructose. The microalgal mixture-supplemented diets were administered for 8 weeks starting 8 weeks after the initiation of the C or H diets. Rats were monitored daily for body weight, food and water intakes. Daily microalgal intake was calculated from the daily food intake. The fatty acid concentrations of both C and H control diets used for the calculation of mean daily fatty acids intake were obtained from our previous study [27].

Abdominal circumference and body length of rats were measured using a standard measuring tape during anaesthesia for systolic blood pressure measurements [26]. Body mass index (BMI) and energy efficiency were calculated [26]. Systolic blood pressure measurements, oral glucose tolerance tests, and insulin tolerance tests were conducted at 0, 8, and 16 wk. All other measurements were made at week 16.

### 2.3. Cardiovascular Measurements

Systolic blood pressure was measured under light sedation following intraperitoneal injection of Zoletil (tiletamine 15 mg/kg, zolazepam 15 mg/kg; Virbac, Milperra, Australia) using a MLT1010 Piezo-Electric Pulse Transducer and inflatable tail-cuff connected to a MLT844 Physiologic Pressure Transducer using PowerLab data acquisition unit (all from ADInstruments, Sydney, Australia) [26].

Echocardiographic examinations (Phillips iE33, 12MHz transducer, Best, The Netherlands) were performed to assess the cardiovascular structure and function in all rats at the end of protocol [26]. Briefly, rats were anaesthetised using Zoletil (intraperitoneal tiletamine 25 mg/kg and zolazepam 25 mg/kg; Virbac, Milperra, Australia) and Ilium Xylazil (intraperitoneal xylazine 15 mg/kg; Troy Laboratories, Smithfield, Australia) and positioned in dorsal recumbencey. Electrodes attached to the skin overlying the elbows and right stifle facilitated the simultaneous recording of a lead II electrocardiogram. A short-axis view of the left ventricle at the level of the papillary muscles was obtained and used to direct acquisition of M mode images of the left ventricle for measurement of wall thicknesses during systole and diastole. Measurements were taken in accordance with the guidelines of the American Society of Echocardiography using the leading-edge method [26] and the ventricular contractility indices were calculated [28,29].

The left ventricular function of the rats in all treatment groups was assessed using the Langendorff heart preparation [26]. Terminal anaesthesia was induced by intraperitoneal injection of pentobarbitone sodium (Lethabarb^®^, 100 mg/kg). After heparin (200 IU; Sigma-Aldrich Australia, Sydney, Australia) administration through the right femoral vein, blood (~5 mL) was taken from the abdominal aorta. Isovolumetric ventricular function was measured by inserting a latex balloon catheter into the left ventricle of the isolated heart connected to a Capto SP844 MLT844 physiologic pressure transducer and Chart software on a MacLab system (both ADInstruments, Sydney, Australia).

Thoracic aortic rings (~4 mm in length) were suspended in an organ bath chamber with a resting tension of approximately 10 mN. Cumulative concentration-response (contraction) curves were measured for noradrenaline (Sigma-Aldrich Australia, Sydney, Australia); concentration-response (relaxation) curves were measured for acetylcholine (Sigma-Aldrich Australia, Sydney, Australia) and sodium nitroprusside (Sigma-Aldrich Australia, Sydney, Australia) in the presence of a submaximal (≈70%) contraction to noradrenaline [26,29].

### 2.4. Oral Glucose Tolerance and Insulin Tolerance Tests

For the oral glucose tolerance test, basal blood glucose concentrations were measured in blood taken from the tail vein of overnight food-deprived rats using Medisense Precision Q.I.D. glucose meter (Abbott Laboratories, Bedford, MA, USA). Fructose-supplemented drinking water in the H and HSC groups was replaced with normal water for the overnight food-deprivation period. The rats were given 2 g/kg body weight of glucose as a 40% aqueous solution via oral gavage. Tail vein blood samples were taken at 30, 60, 90 and 120 min following glucose administration. For insulin tolerance test, basal blood glucose concentrations were measured after 4–5 h of food deprivation as above. The rats were injected intraperitoneally with 0.33 IU/kg insulin-R (Eli-Lilly Australia, West Ryde, Australia) and tail vein blood samples were collected at 0, 15, 30, 45, 60, 90 and 120 min. Rats were withdrawn from the test if the blood glucose concentration dropped below 1.1 mmol/L, and 4 g/kg glucose was administered immediately via oral gavage to prevent hypoglycaemia.

### 2.5. Body Composition Measurements

Dual energy X-ray absorptiometric (DXA) measurements were performed on rats after 16 weeks of feeding. This was done 2 days before rats were euthanised for pathophysiological assessments, using a Norland XR36 DXA instrument (Norland Corp., Fort Atkinson, WI, USA). DXA scans were analysed using the manufacturer’s recommended software for use in laboratory animals (Small Subject Analysis Software, version 2.5.3/1.3.1, Norland Corp., Fort Atkinson, WI, USA) [25,26]. The precision error of lean mass for replicate measurements, with repositioning, was 3.2%. Visceral adiposity index (%) was calculated [25,26].

### 2.6. Organ Weights

The right and left ventricles were separated after perfusion experiments and weighed. Liver and retroperitoneal, epididymal and omental fat pads were removed following heart removal and blotted dry for weighing. Organ weights were normalised relative to the tibial length at the time of their removal (in mg/mm).

### 2.7. Histology

Two rats per group were taken exclusively for histological analysis. Two slides were prepared per tissue specimen and two random, non-overlapping fields per slide were taken. Organs were also collected from rats used for perfusion studies. Immediately after removal, heart and liver tissues were fixed in 10% neutral buffered formalin for 3 days and then dehydrated and embedded in paraffin wax [25,26]. Thin sections (7 μm) of left ventricle and the liver were cut and stained with haematoxylin and eosin for determination of inflammatory cell infiltration and fat vacuole enlargement with 20× objectives using an Olympus BX51 microscope (Olympus, Melville, NY). Collagen distribution was measured in the left ventricle with picrosirius red stain with laser confocal microscopy (Zeiss LSM 510 upright Confocal Microscope, Carl Zeiss, North Ryde, Australia) [26,29].

### 2.8. Plasma Biochemistry

Blood was centrifuged at 5000× *g* for 15 min within 30 min of collection into heparinised tubes. Plasma was separated and transferred to Eppendorf tubes (Thermo Fisher Scientific, Scoresby, Australia) for storage at −20 °C before analysis. Activities of plasma enzymes and analyte concentrations were determined using kits and controls supplied by Olympus using an Olympus analyser (AU 400, Olympus, Tokyo, Japan) [26,29].

### 2.9. Statistical Analyses

All data are presented as mean ± SEM. Data from C, CSC, H and HSC groups were compared in a series of two-way ANOVAs with “Diet” as the fixed factor, either high carbohydrate and high fat diet or control corn starch diet and “Treatment”, diet supplemented with or without microalgae, as the fixed factors. Homogeneity of variance for ANOVA was assessed using Bartlett's test and variables that were not normally distributed were log-transformed prior to analysis. Where the main effects were significant (*p* < 0.05), means were compared using Newman-Keuls multiple comparisons. Where transformations did not result in normality or homogeneity of variance, a Kruskal-Wallis non-parametric test was performed. All statistical analyses were run using GraphPad Prism version 5.00 for Windows (GraphPad Software, La Jolla, CA, USA).

## 3. Results

### 3.1. Nutritional Composition of Microalgal Mixture and Intake of Essential Mineral Ions and Fatty Acids

In the SC mixture, total protein content was 46.1% of dry algae, total fibre content was 19.6% of dry algae with <0.1% as soluble fibre, total fatty acid content was 4.84% of dry algae with 86% of polyunsaturated fatty acids (PUFA) as omega-3 fatty acids and 14% of PUFA as omega-6 fatty acids, and mineral ion content was 3.7% of dry algae, including, in g/100g dry weight, magnesium 0.44, potassium 0.90, sodium 0.53, calcium 0.27 and iron 0.27.

SC supplementation in CSC and HSC rats increased protein and fibre intake, and magnesium (38.2 mg/day), sodium (55.6 mg/day), potassium (181.1 mg/day), and zinc (0.96 mg/day) intake in CSC compared to C rats, while the HSC rats had higher zinc intake (0.52 mg/day) compared to the H rats (0.38 mg/day) (Table 1). Compared to C rats, the H rats showed increased fat intake including saturated fatty acids, mono-unsaturated fatty acids and PUFA (Table 1). SC supplementation did not change the total fatty acids intake in either CSC or HSC rats, while increased intake of α-linolenic acid (ALA) was measured in CSC and HSC rats (Table 1). CSC rats showed increased EPA and DHA intake compared to HSC rats (Table 1).

### 3.2. Physiology and Metabolic Variables

**Figure 1:**
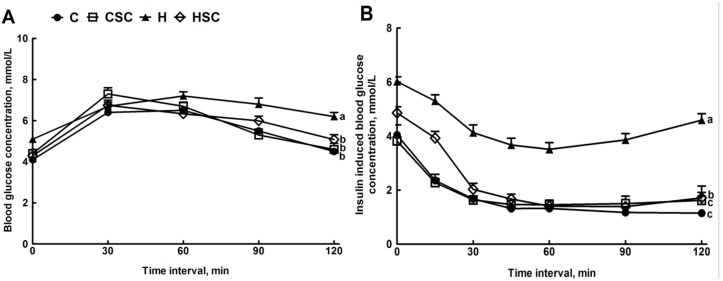
Effect of microalgal mixture treatment on oral glucose tolerance (**A**) and insulin tolerance (**B**) in C, CSC, H and HSC rats. Values are mean ± SEM, *n* = 10. Endpoints means without a common letter differ significantly at *p* < 0.05. C, corn starch-fed rats; CSC, cornstarch rats treated with *Scenedesmus dimorphus + Schroederiella apiculata*; H, high carbohydrate, high fat diet-fed rats; HSC, high carbohydrate, high fat-rats treated with *Scenedesmus dimorphus + Schroederiella apiculata*.

The food for the C rats had a much lower energy intake at 11.4 kJ/g than the food for H rats at 21.4 kJ/g. Food and water consumption were higher in C rats compared with H rats (Table 2). Compared with C rats, increased energy intake, body weight gain, energy efficiency, total body fat mass, abdominal fat mass, abdominal circumference and visceral adiposity were observed in H rats (Table 2). HSC rats showed lowered total body fat mass and total abdominal fat mass with decreased abdominal circumference and visceral adiposity index (Table 2). The glucose tolerance and insulin sensitivity were improved in HSC rats, compared with H rats with no change in C and CSC (Figure 1; Table 2). The bone mineral content was higher in H rats and reduced in HSC rats (Table 2). Increased plasma concentrations of NEFA, triglycerides and total cholesterol were observed in H rats, whereas no changes were observed in CSC rats (Table 2). SC treatment normalised the plasma concentrations of NEFA, triglycerides and total cholesterol in HSC rats (Table 2). No changes in plasma potassium and magnesium ion concentrations were observed in the treatment groups, while SC treatment lowered plasma sodium concentrations in C and H rats (Table 2).

**Table 1:** Protein, fibre, mineral ion and fat intake in C, H, CSC and HSC rats.

Variable	C	CSC	H	HSC	*p*-Value
					Diet	Treatment	Interaction
Total protein, *g/day*	0.99 ± 0.05 ^b^	1.86 ± 0.14 ^a^	1.22 ± 0.08 ^b^	1.75 ± 0.09 ^a^	0.54	<0.0001	0.08
Total fibre, *mg/day*	231.3 ± 12.2 ^c^	584.5 ± 42.4 ^a^	154.8 ± 9.6 ^d^	369.5 ± 20.4 ^b^	<0.0001	<0.0001	0.009
Magnesium intake, *mg/day*	28.6 ± 1.5 ^b^	38.2 ± 2.8 ^a^	14.4 ± 0.9 ^c^	19.3 ± 1.1 ^c^	<0.0001	0.0002	0.19
Potassium intake, *mg/day*	148.7 ± 7.8 ^b^	181.1 ± 13.1 ^a^	93.1 ± 5.8 ^c^	105.3 ± 5.8 ^c^	<0.0001	0.016	0.26
Sodium intake, *mg/day*	46.1 ± 2.4 ^b^	55.6 ± 4.0 ^a^	27.9 ± 1.7 ^c^	32.1 ± 1.8 ^c^	<0.0001	<0.015	0.33
Calcium intake, *mg/day*	225 ± 12 ^a^	249 ± 18 ^a^	163 ± 10 ^b^	170 ± 9 ^b^	<0.0001	0.24	0.52
Zinc intake, *mg/day*	0.79 ± 0.01 ^b^	0.96 ± 0.03 ^a^	0.38 ± 0.03 ^b^	0.52 ± 0.01 ^a^	<0.0001	<0.0001	0.52
Total fat intake, *g/day*	0.25 ± 0.01 ^b^	0.36 ± 0.03 ^b^	5.01 ± 0.31 ^a^	5.19 ± 0.29 ^a^	<0.0001	0.49	0.87
Saturated fatty acid, *g/day*	0.07 ± 0.00 ^b^	0.09. ± 0.01 ^b^	2.66 ± 0.16 ^a^	2.74 ± 0.15^a^	<0.0001	0.65	0.78
MUFA, *g/day*	0.09 ± 0.01 ^b^	0.11 ± 0.01 ^b^	2.21 ± 0.14 ^a^	2.27 ± 0.13 ^a^	<0.0001	0.67	0.83
PUFA, *g/day*	0.09 ± 0.01 ^c^	0.15 ± 0.01 ^b^	0.14 ± 0.01 ^b^	0.18 ± 0.01 ^a^	0.0003	<0.0001	0.33
ALA, *mg/day*	11.6 ± 0.6 ^d^	42.1 ± 3.1 ^a^	4.8 ± 0.3 ^c^	23.6 ± 1.3 ^b^	<0.0001	<0.0001	0.002
EPA, *mg/day*	0.00 ± 0.00 ^c^	0.28 ± 0.02 ^a^	0.00 ± 0.00 ^c^	0.18 ± 0.01^b^	<0.0001	<0.0001	<0.0001
DHA, *mg/day*	0.00 ± 0.00 ^c^	0.36 ± 0.03 ^a^	0.00 ± 0.00 ^c^	0.23 ± 0.01 ^b^	<0.0001	<0.0001	<0.0001

Values are mean ± SEM, *n* = 9–10. Means within a row without a common letter significantly differ at *p* < 0.05. C, corn starch-fed rats; CSC, corn starch-rats treated with *Scenedesmus dimorphus + Schroederiella apiculata*; H, high carbohydrate, high fat diet-fed rats; HSC, high carbohydrate, high fat-rats treated with *Scenedesmus dimorphus + Schroederiella apiculata.*

**Table 2:** Metabolic and biochemical variables in C, H, CSC and HSC rats.

Variable	C	CSC	H		HSC	*p*-Value		
						Diet	Treatment	Interaction
Food intake, *g/day*	31.3 ± 1.6 ^a^	34.2 ± 2.5 ^a^	20.9 ± 1.3 ^b^		21.5 ± 1.2 ^b^	<0.0001	0.32	0.51
Water intake, *mL/day*	31.3 ± 1.8 ^a^	32.6 ± 2.3 ^a^	19.0 ± 1.4 ^b^		20.9 ± 1.5 ^b^	<0.0001	0.38	0.87
Energy intake, *kJ/day*	356.3 ± 2.4 ^d^	417.9 ± 10.8 ^c^	440.0 ± 5.1 ^b^		475.4 ± 5.1 ^a^	<0.0001	<0.0001	0.06
Feed conversion efficiency, *%*	2.0 ± 0.2 ^c^	2.0 ± 0.2 ^c^	4.5 ± 0.3 ^a^		3.3 ± 0.3 ^b^	<0.0001	0.024	0.024
Body weight gain (8–16 weeks), *%*	7.2 ± 0.9 ^c^	8.6 ± 1.0 ^c^	19.7 ± 1.2 ^a^		15.5 ± 1.5 ^b^	0.002	0.001	0.144
Bone mineral content, *g*	12.4 ± 0.4 ^c^	11.8 ± 0.2 ^c^	16.2 ± 0.5 ^a^		13.9 ± 0.4 ^b^	<0.0001	0.0009	0.038
Total fat mass, *g*	97.4 ± 6.7 ^c^	63.4 ± 6.9 ^d^	197.8 ± 14.1 ^a^		129.1 ± 10.4 ^b^	<0.0001	<0.0001	0.094
Total lean mass, *g*	287.0 ± 3.9 ^b^	320.7 ± 6.6 ^a^	279.1 ± 10.9 ^b^		317.3 ± 7.1 ^a^	0.621	0.059	0.360
Abdominal circumference, *cm*	18.2 ± 0.1 ^c^	18.7 ± 0.2 ^c^	22.2 ± 0.3 ^a^		20.5 ± 0.1 ^b^	<0.0001	0.0038	<0.0001
Tissue wet weights, *mg/mm tibial length*								
Retroperitoneal fat	119.0 ± 9.0 ^c^	121.8 ± 12.7 ^c^	365.3 ± 26.3 ^a^	235.6 ± 13.1 ^b^		<0.0001	<0.0001	0.0020
Epididymal fat	92.8 ± 7.5 ^c^	100.9 ± 7.8 ^c^	208.2 ± 14.3 ^a^	133.7 ± 11.9 ^b^		<0.0001	0.0039	0.0005
Omental fat	60.0 ± 5.0 ^b^	75.3 ± 8.1 ^b^	125.3 ± 10.6 ^a^	70.9 ± 5.9 ^b^		0.0003	0.016	<0.0001
Total abdominal fat	271.8 ± 19.4 ^c^	298.1 ± 27.8 ^c^	698.8 ± 47.2 ^a^	440.2 ± 28.4 ^b^		<0.0001	0.0010	<0.0001
Visceral adiposity index, *%*	3.3 ± 0.2 ^c^	3.5 ± 0.3 ^c^	6.6 ± 0.5 ^a^	4.7 ± 0.4 ^b^		<0.0001	0.027	0.007
Plasma NEFA, *mmol/L*	1.50 ± 0.08 ^b^	1.89 ± 0.08 ^b^	4.16 ± 0.12 ^a^	1.62 ± 0.18 ^b^		<0.0001	<0.0001	<0.0001
Glucose AUC, *mmol/L/120 minutes*	681 ± 14 ^b^	715 ± 25 ^b^	804 ± 21 ^a^	713 ± 14^b^		0.002	0.13	0.003
Insulin AUC, *mmol/L/120 minutes*	146 ± 17 ^c^	157 ± 24 ^c^	505 ± 28 ^a^	237 ± 13 ^b^		<0.0001	<0.0001	<0.0001
Plasma triglycerides, *mmol/L*	0.51 ± 0.07^b^	0.56 ± 0.03 ^b^	2.69 ± 0.04 ^a^	0.39 ± 0.05 ^b^		<0.0001	<0.0001	<0.0001
Plasma total cholesterol, *mmol/L*	1.48 ± 0.06 ^b^	1.64 ± 0.03 ^b^	2.01 ± 0.05 ^a^	1.64 ± 0.04 ^b^		<0.0001	0.032	<0.0001
Plasma Na^+^, *mmol/L*	142.3 ± 0.4 ^a^	140.0 ± 0.5 ^b^	142.8 ± 0.4 ^a^	140.0 ± 0.2 ^b^		0.53	<0.0001	0.53
Plasma K^+^, *mmol/L*	5.43 ± 0.39	5.70 ± 0.26	6.28 ± 0.78	6.95 ± 0.58		0.06	0.39	0.71
Plasma Mg^2+^, *mmol/L*	0.75 ± 0.01	0.81 ± 0.04	0.75 ± 0.03	0.80 ± 0.02		0.86	0.05	0.86

Values are mean ± SEM, *n* = 9–10. Mean within a row without a common letter significantly differ at *p* < 0.05. C, corn starch-fed rats; CSC, corn starch-rats treated with *Scenedesmus dimorphus + Schroederiella apiculata*; H, high carbohydrate, high fat diet-fed rats; HSC, high carbohydrate, high fat-rats treated with *Scenedesmus dimorphus + Schroederiella apiculata.*

### 3.3. Cardiovascular Changes

**Figure 2:**
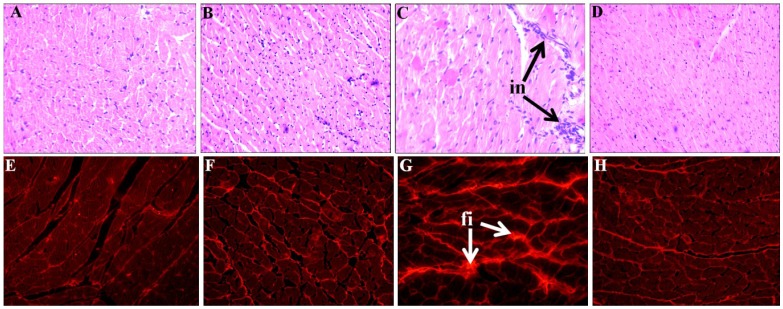
Effect of microalgae mixture treatment on inflammation and fibrosis in the heart. Haematoxylin and eosin staining of left ventricle showing infiltration of inflammatory cells (**A**–**D**, inflammatory cells marked as “in”) (20×) from C (**A**), CSC (**B**), H (**C**), and HSC (**D**). Picrosirius red staining of left ventricle showing collagen deposition (**E**–**H**), fibrosis marked as “fi”) (20×) from C (**E**), CSC (**F**), H (**G**), and HSC (**H**) rats. C, corn starch fed rats; CSC, cornstarch rats treated with *Scenedesmus dimorphus + Schroederiella apiculata*; H, high carbohydrate, high fat diet fed rats; HSC, high carbohydrate, high fat rats treated with *Scenedesmus dimorphus + Schroederiella apiculata*.

**Figure 3:**
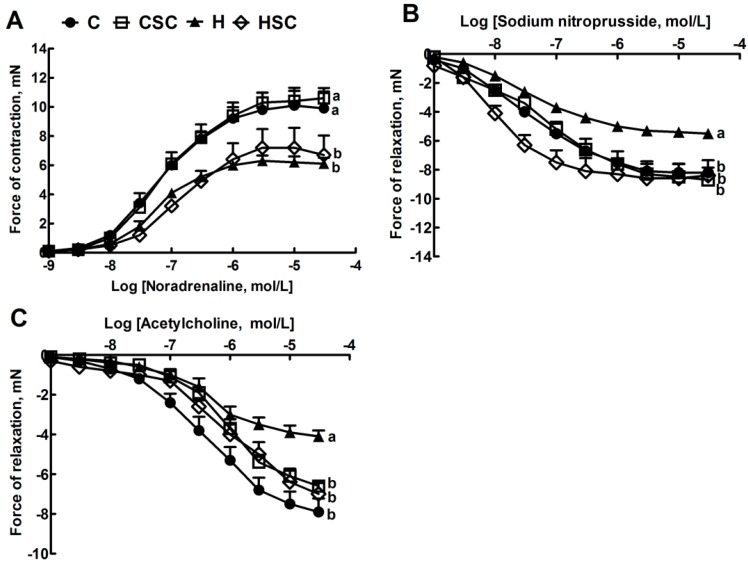
Effect of microalgae mixture treatment on noradrenaline-induced contraction (**A**), sodium nitroprusside-induced relaxation (**B**); and acetylcholine-induced relaxation (**C**) in thoracic aortic preparations from C, CSC, H and HSC rats. Values are mean ± SEM, *n* = 9–10. Endpoints means without a common letter differ significantly at *p* < 0.05. C, corn starch-fed rats; CSC, corn starch rats treated with *Scenedesmus dimorphus + Schroederiella* *apiculata*; H, high carbohydrate, high fat diet-fed rats; HSC, high carbohydrate, high fat rats treated with *Scenedesmus dimorphus + Schroederiella apiculata.*

Systolic blood pressure was unchanged in C groups while the increased systolic blood pressure in H rats was lowered in HSC rats (Table 3). The ratio of ESS:LVIDs was higher in H rats compared to C groups, while no change was observed in SBP:LVIDs and SBP:systolic volume ratios (Table 3). The ESS:LVIDs ratio as an index of ventricular contractility was normalised in HSC rats (Table 3). Further, no change in cardiac structure and function was observed in the microalgal treatment groups compared to C or H rats (Table 3). The left ventricular septum weights and heart weights were increased in H rats compared to C rats (Table 3). SC mixture normalised the left ventricle + septum weight and total heart wet weight in HSC rats (Table 3). Left ventricle showed increased infiltration of inflammatory cells (Figure 2C) and interstitial collagen deposition (Figure 2G) in H rats, compared with C rats (Figure 2A,B and E,F). In HSC rats, the infiltration of inflammatory cells (Figure 2D) and the interstitial collagen deposition (Figure 2H) were normalised. The diastolic stiffness constant (κ) was normalised in HSC rats, compared with H rats (Table 3). Lower contractile responses to noradrenaline in isolated thoracic rings were measured in H and HSC rats compared to C and CSC groups (Figure 3A). Lower smooth muscle-dependent and endothelium-dependent relaxant responses to sodium nitroprusside and acetylcholine were measured in H rats (Figure 3B,C); both responses were higher in HSC rats (Figure 3B,C).

**Table 3:** Cardiovascular variables in C, H, CSC and HSC rats.

Variable	C	CSC	H	HSC	*p*-Value		
					Diet	Treatment	Interaction
SBP, *mmHg*	125 ± 2 ^c^	130 ± 1 ^c^	152 ± 2 ^a^	138 ± 2 ^b^	<0.0001	0.08	0.0005
Heart rate	304.5 ± 30.5 ^ab^	241.4 ± 20.6 ^b^	332.5 ± 14.1 ^a^	372.0 ± 20.0 ^a^	0.0013	0.60	0.028
LVIDd, *mm*	7.1 ± 0.3	7.6 ± 0.1	7.8 ± 0.2	7.2 ± 0.1	0.45	0.80	0.008
LVIDs, *mm*	3.7 ± 0.4	4.1 ± 0.2	3.7 ± 0.2	3.4 ± 0.2	0.20	0.85	0.20
LVPWd, *mm*	1.72 ± 0.04 ^b^	1.81 ± 0.04 ^b^	2.03 ± 0.04 ^a^	1.83 ± 0.04 ^b^	0.0006	0.27	0.0006
LVPWs, *mm*	2.70 ± 0.10	2.99 ± 0.08	3.10 ± 0.08	3.06 ± 0.12	0.187	0.087	0.614
IVSd, *mm*	1.87 ± 0.09	1.86 ± 0.07	2.04 ± 0.03	1.91 ± 0.03	0.08	0.26	0.33
IVSs, *mm*	2.97 ± 0.15	3.09 ± 0.08	3.47 ± 0.16	3.13 ± 0.13	0.05	0.42	0.09
Diastolic volume, μL	378.0 ± 43.7	467.0 ± 24.8	493.0 ± 42.5	385.0 ± 17.9	0.63	0.78	0.007
Systolic volume, μL	64.0 ± 15.9	76.0 ± 8.9	57.0 ± 7.7	44.0 ± 6.7	0.07	0.96	0.24
SBP:LVIDs	36.6 ± 3.8	32.5 ± 1.7	40.9 ± 1.8	37.9 ± 1.9	0.06	0.16	0.82
SBP:systolic volume	3482 ± 983	1982 ± 314	3016 ± 418	3328 ± 451	0.47	0.33	0.14
ESS:LVIDs	2.02 ± 0.07 ^b^	2.21 ± 0.06 ^b^	2.44 ± 0.08 ^a^	2.11 ± 0.09 ^b^	0.044	0.36	0.002
Stroke volume, μL	315 ± 31 ^b^	392 ± 21 ^ab^	436 ± 38 ^a^	340 ± 14 ^b^	0.22	0.73	0.004
Cardiac output, *mL/min*	95.2 ± 10.8 ^b^	94.8 ± 9.9 ^b^	142.6 ± 9.6 ^a^	125.9 ± 6.7 ^ab^	0.0003	0.37	0.39
Relative wall thickness	0.51 ± 0.03	0.48 ± 0.02	0.53 ± 0.02	0.52 ± 0.01	0.17	0.35	0.64
Systolic wall stress	88.6 ± 8.4	91.5 ± 5.7	91.4 ± 5.7	72.8 ± 5.8	0.23	0.24	0.11
Fractional shortening, %	47.8 ± 3.5	48.2 ± 1.2	52.0 ± 1.6	52.4 ± 1.8	0.07	0.86	1.00
Ejection fraction	84.5 ± 2.7	85.4 ± 1.3	88.5 ± 1.1	88.7 ± 1.3	0.043	0.75	0.84
Estimated LV mass, *g*	0.90 ± 0.07 ^b^	1.09 ± 0.06 ^ab^	1.24 ± 0.06 ^a^	0.97 ± 0.04 ^b^	0.07	0.50	0.0005
LV+septum wet weight, *mg/mm*	17.3 ± 0.4 ^b^	18.1 ± 0.6 ^b^	20.0 ± 0.6 ^a^	17.2 ± 0.5 ^b^	0.10	0.07	0.002
RV wet weight, *mg/mm*	2.3 ± 0.1 ^bc^	3.0 ± 0.2 ^a^	4.0 ± 1.2 ^bc^	2.0 ± 0.1 ^bc^	0.57	0.30	0.036
Heart wet weight, *mg/mm*	19.6 ± 0.4 ^b^	21.1 ± 0.6 ^b^	24.0 ± 1.6 ^a^	19.2 ± 0.5 ^b^	0.18	0.08	0.0014
LV+septum wet weight, *mg/mm*	17.3 ± 0.4 ^b^	18.1 ± 0.6 ^b^	20.0 ± 0.6 ^a^	17.2 ± 0.5 ^b^	0.10	0.07	0.002
Diastolic stiffness, *κ*	23.8 ± 0.7 ^b^	22.8 ± 0.5 ^b^	29.8 ± 2.2 ^a^	24.8 ± 1.1 ^b^	0.005	0.029	0.14

Values are mean ± SEM, *n* = 8–10. Mean within a row without a common letter significantly differ at *p* < 0.05. C, corn starch-fed rats; CSC, corn starch-rats treated with *Scenedesmus dimorphus + Schroederiella apiculata*; H, high carbohydrate, high fat diet-fed rats; HSC, high carbohydrate, high fat-rats treated with *Scenedesmus dimorphus + Schroederiella apiculata*; LVID, left ventricular diameter in diastole (d) or systole (s); LVPW, left ventricular posterior wall thickness in diastole (d) or systole (s); IVS, interventricular septum thickness in diastole (d) or systole (s); SBP, systolic blood pressure; ESS, end-systolic stress.

### 3.4. Hepatic Structure and Function

**Figure 4:**
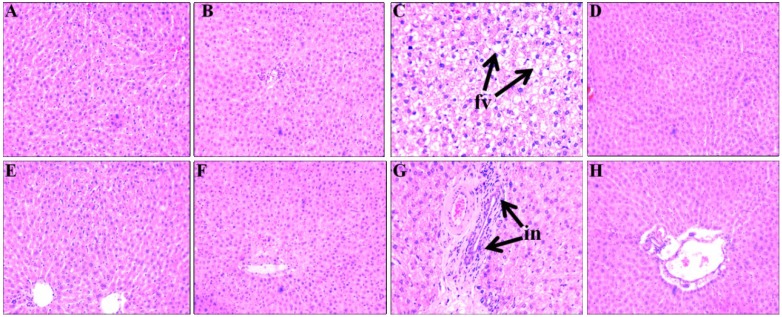
Effect of microalgae mixture treatment on inflammation and fat deposition in the liver. Haematoxylin and eosin staining of liver showing enlarged fat vacuoles (**A**–**D**, marked as “fv”) (20×) and inflammatory cells (**E**–**H**, marked as “in”) (20×) from C (**A**,**E**), CSC (**B**,**F**), H (**C**,**G**), HSC (**D**,**H**). C, corn starch-fed rats; CSC, corn starch rats treated with *Scenedesmus dimorphus* +*Schroederiella apiculata*; H, high carbohydrate, high fat diet-fed rats; HSC, high carbohydrate, high fat rats treated with *Scenedesmus dimorphus* + *Schroederiella apiculata*.

Compared to C groups, H rats showed increased liver weight (Table 4), higher infiltration of inflammatory cells and presence of enlarged fat vacuoles (Figure 4A–C & E–G). SC treatment prevented the infiltration of inflammatory cells in HSC treated rats (Figure 4H). Liver weight was normalised in HSC rats compared to H rats (Table 4). Further, hepatocytes with enlarged fat vacuoles were not observed in HSC rats (Figure 4D). Plasma activities of liver enzymes ALT and AST were higher in H rats compared to C rats whereas both the ALT and AST activities were normalised in HSC rats (Table 4).

**Table 4:** Liver variables in C, H, CSC and HSC rats.

Variable	C	CSC	H	HSC	*p*-Value		
					Diet	Treatment	Interaction
Liver weight	239.3 ± 4.4 ^b^	221.8 ± 4.3 ^b^	337.5 ± 9.8 ^a^	226.1 ± 6.1 ^b^	<0.0001	<0.0001	<0.0001
Plasma ALT activity, U/L	27.5 ± 2.5 ^b^	26.6 ±2.9 ^b^	44.4 ± 2.4 ^a^	22.7 ± 1.8 ^b^	0.012	<0.0001	0.0002
Plasma AST activity, U/L	70.8 ± 3.5 ^b^	70.5 ± 9.3 ^b^	103.3 ± 5.6 ^a^	78.4 ± 2.9 ^b^	0.002	0.041	0.046

Values are mean ± SEM, *n* = 9–10. Mean within a row without a common letter significantly differ at *p* < 0.05. C, corn starch-fed rats; CSC, corn starch rats treated with *Scenedesmus dimorphus + Schroederiella apiculata*; H, high carbohydrate, high fat diet-fed rats; HSC, high carbohydrate, high fat rats treated with *Scenedesmus dimorphus + Schroederiella apiculata.*

## 4. Discussion

Consumption of high-energy diets with increased saturated or *trans* fatty acids and refined carbohydrates initiates body weight gain and visceral obesity [30,31,32]. In our study, an increased energy intake led to the metabolic changes associated with metabolic syndrome, together with cardiovascular remodelling. The microalgal SC mixture contains many potential bioactive compounds, including minerals and omega-3 fatty acids. However, the doses of these compounds, either together or individually, in this study appear inadequate to reverse the H diet-induced metabolic changes based on previous studies [27,33,34]. In contrast, the doses of insoluble fibre and possibly protein may be sufficient to produce therapeutic responses.

SC supplementation increased insoluble fibre intake to 369.5–584.5 mg/day (Table 1) and reduced body weight in HSC rats. This dose seems sufficient to prevent the metabolic syndrome in H rats, based on comparison with human data. Based on body surface area, the human dose of daily dietary fibre intake from this dose in rats can be calculated as 8.3–13.1 g/day [35], which is similar to the mean dietary fibre intake (13.1–16.1 g/day) of the US population for 1999–2008 [36], and to the dietary fibre intake (7.2–9.3 g/day) of weight loss diets such as the South Beach diet and Atkins diet [37]. Further, dietary fibre intake (7 g/day) reduced body weight by 5 kg in overweight patients [38]. Insoluble fibre in SC treatment may enhance the fecal energy loss by fecal bulking effect in rats, humans and mice [39,40,41], and therefore possibly decrease the fat digestibility [40,42]. The increased lean mass detected in HSC rats suggests an increased skeletal muscle β-oxidation of fatty acids possibly induced by the insoluble fibre component of SC mixture [43] that may enhance the energy expenditure and fat-free mass development [44]. Dysregulation of adipose-derived adipokines and NEFA toxicity support abdominal obesity as a key factor mediating metabolic and cardiovascular disease [45]. Thus, decreased abdominal obesity in HSC rats may be the driver for the improvement of all signs of the metabolic syndrome. Additionally, increased lean mass may contribute to the improved glucose utilisation and insulin sensitivity in HSC rats.

Insoluble fibre as in SC may also act as a prebiotic to change the gut microbiome and therefore modify obesity [46,47]. This assumes that the SC insoluble fibre can be metabolised in the colon to short chain fatty acids that are substrates for beneficial microbacteria, thus improving gut health.

Further, seaweeds may provide sufficient protein to improve obesity [47]. The dose of protein in this study in rats can be calculated as 0.57–0.60 g/kg body weight/day in humans, similar to the recommended dietary allowance of total protein of 0.8 g/kg body weight/day in the management of type 2 diabetes [48]. High protein diets are controversial as weight loss protocols but a meta-analysis showed beneficial effects on weight loss, glycated haemoglobin concentrations and blood pressure with these diets [49].

The relevance of these animal model studies to human nutrition may be questioned. The model has been well characterised as a model of metabolic syndrome [25,26,27,29,50]. Nevertheless, the diets differ in macronutrient composition with the H diet containing more carbohydrates (68%/diet as fructose and sucrose) and fat (23.9%/diet as mono-unsaturated and saturated fatty acids) serving as the major sources of energy with 21.4 kJ/g, compared to the low fat (8% of diet)-C diet which contains an increased proportion of slow digestible carbohydrates (also 68%/diet) providing 11.4 kJ/g. However, variation in diet is characteristic of human nutrition and a large body of evidence exists demonstrating that simple variation in macronutrient distribution in the diet can provide positive benefits, for example improved body composition, in metabolic syndrome (see [51] for a review). More studies are needed to show whether the proteins present in the SC mixture have therapeutic responses on the signs of the metabolic syndrome.

Vascular disease is increased with increased ectopic fat in humans [52]. Visceral obesity associated hypertension, hyperglycaemia, insulin resistance and dyslipidaemia increase the arterial stiffness leading to endothelial dysfunction [53]. Reduction in abdominal obesity in HSC rats was associated with an improved cardiac structure and function, diminished vasoconstriction, and improved endothelial and vascular smooth muscle function. Further, SC supplementation prevented infiltration of inflammatory cells in the heart, attenuated the interstitial ventricular collagen deposition and decreased cardiac stiffness in HSC rats; the links between adipokines, inflammation and cardiovascular disease are well described [54]. SC treatment also reduced inflammatory cell infiltration in the liver tissue and improved the liver morphology and function of HSC rats, probably related to the decreased visceral adiposity and therefore adipokine production as well as an improved plasma lipid profile [55].

## 5. Conclusions

This study suggests that the supplementation of insoluble fibre and possibly protein from SC mixture increases the lean mass, prevents fat deposition especially visceral fat and can normalise the impaired glucose and insulin tolerance, hypertension, endothelial dysfunction, collagen deposition and cardiac stiffness, inflammatory cell infiltration of heart and liver, and non-alcoholic fatty liver disease in diet-induced obese rats. Further characterisation of insoluble fibre and protein present in SC should be carried out to define the most active components given the impacts of the microalgal mixture on the wide range of signs of the metabolic syndrome induced by obesity.
